# Insulin Resistance Associated with Plasma Xanthine Oxidoreductase Activity Independent of Visceral Adiposity and Adiponectin Level: MedCity21 Health Examination Registry

**DOI:** 10.1155/2019/1762161

**Published:** 2019-12-30

**Authors:** Masafumi Kurajoh, Shinya Fukumoto, Takayo Murase, Takashi Nakamura, Takuma Ishihara, Hirofumi Go, Kouji Yamamoto, Shinya Nakatani, Akihiro Tsuda, Tomoaki Morioka, Katsuhito Mori, Yasuo Imanishi, Masaaki Inaba, Masanori Emoto

**Affiliations:** ^1^Department of Metabolism, Endocrinology, and Molecular Medicine, Osaka City University Graduate School of Medicine, Osaka, Japan; ^2^Department of Premier Preventive Medicine, Osaka City University Graduate School of Medicine, Osaka, Japan; ^3^Mie Research Laboratories, Sanwa Kagaku Kenkyusho Co., Ltd., Inabe, Mie, Japan; ^4^Innovative and Clinical Research Promotion Center, Gifu University Hospital, Gifu, Japan; ^5^Department of Medical Statistics, Osaka City University Graduate School of Medicine, Osaka, Japan; ^6^Department of Biostatistics, Yokohama City University School of Medicine, Yokohama, Japan; ^7^Department of Nephrology, Osaka City University Graduate School of Medicine, Osaka, Japan

## Abstract

**Background:**

Higher levels of uric acid production have been reported in individuals with visceral fat obesity, and obesity is known to enhance xanthine oxidoreductase (XOR) activity, although the precise mechanism remains unclear. We investigated the associations of visceral fat area (VFA), serum adiponectin level, and insulin resistance with plasma XOR activity using our novel highly sensitive assay based on [^13^C_2_,^15^N_2_] xanthine and liquid chromatography/triple quadrupole mass spectrometry.

**Methods:**

This cross-sectional study included 193 subjects (92 males and 101 females) registered in the MedCity21 health examination registry. Plasma XOR activity, serum adiponectin level, and VFA obtained by computed tomography were measured, and insulin resistance was determined based on the homeostasis model assessment (HOMA-IR) index.

**Results:**

The mean values for VFA, log HOMA-IR, and log plasma XOR activity were 76.8 ± 45.8 cm^2^, 0.14 ± 0.30, and 1.50 ± 0.44 pmol/h/mL, respectively. Multiple regression analysis showed that HOMA-IR was significantly (*p*=0.020) associated with plasma XOR activity independent of other factors, including VFA and adiponectin level, as well as age, sex, alcohol drinking habit, smoking habit, alanine transaminase, HbA1c, and eGFR. The “*sex∗HOMA* − IR” interaction was not significant (*p*=0.020) associated with plasma XOR activity independent of other factors, including VFA and adiponectin level, as well as age, sex, alcohol drinking habit, smoking habit, alanine transaminase, HbA1c, and eGFR. The “

**Conclusions:**

Our results indicate that insulin resistance is associated with plasma XOR activity and that relationship is independent of visceral adiposity and adiponectin level, suggesting that the development of insulin resistance resulting from increased visceral adiposity and/or reduced serum adiponectin contributes to increased uric acid production by stimulating XOR activity.

## 1. Introduction

Hyperuricemia is frequently complicated with obesity in individuals with visceral fat accumulation [1, 2], which is strongly associated with insulin resistance [3]. In affected patients, hyperuricemia is considered to be caused by reduced renal excretion of uric acid as a consequence of insulin-mediated renal reabsorption of uric acid in the proximal tubules, as a result of insulin resistance [4]. However, previous studies have emphasized that individuals with visceral fat obesity also have increased production of uric acid [2, 5].

Xanthine oxidoreductase (XOR) is a rate-limiting enzyme for *in vivo* uric acid production that catalyzes oxidation not only from hypoxanthine to xanthine but also from xanthine to uric acid in the purine metabolism pathway [6]. In humans, XOR is exclusively expressed in the liver and intestines but not in adipose tissue [7, 8]; thus, elevated plasma XOR activity in obese subjects can be explained by increased XOR activity in the liver and intestines but not in adipose tissue [9, 10]. Furthermore, those reports suggested that visceral fat accumulation might indirectly contribute to increased XOR activity preferentially in the liver but not intestines, resulting in overproduction of uric acid, although the precise mechanism remains unclear. Of importance, some studies have shown an association of insulin resistance with plasma XOR activity in young healthy subjects, a general population cohort, and subjects with familial combined hyperlipidemia [11–13].

To the best of our knowledge, no studies have examined the association of insulin resistance with plasma XOR activity together with adiposity and adipocytokines. The purpose of the present study was to examine the associations of visceral fat area (VFA), obtained by computed tomography (CT), as well as serum adiponectin level and insulin resistance, assessed by homeostatic model assessment of insulin resistance (HOMA-IR), a reliable surrogate marker for insulin resistance [14, 15], with plasma XOR activity using our newly developed assay for determining XOR activity [16, 17] in subjects who participated in the MedCity21 health examination registry.

## 2. Materials and Methods

### 2.1. Study Design

The MedCity21 health examination registry was instituted from April 2015 in a comprehensive manner to elucidate the causes of various diseases occurring in adults (cancer, diabetes mellitus (DM), cardiovascular disease, cerebrovascular disease, mental disorders, dyslipidemia, hypertension, hyperuricemia, obesity, chronic respiratory disease, liver disease, digestive disease, gynecological diseases, skin disease, etc.) for the development of advanced diagnostic techniques, treatment methods, and prevention methods for patients with those diseases [18–20]. Individuals who underwent comprehensive medical examinations at MedCity21 at the Osaka City University Hospital Advanced Medical Center for Preventive Medicine (Osaka, Japan) were registered. The MedCity21 health examination registry protocol was approved by the Ethics Committee of Osaka City University Graduate School of Medicine (approval No. 2927). Written informed consent was obtained from all subjects, and the study was conducted in full accordance with the Declaration of Helsinki. The present study protocol was approved by the Ethics Committee of Osaka City University Graduate School of Medicine (approval No. 3684) and performed with an opt out option, explained in instructions on the website of the hospital. Using findings presented in the MedCity21 health examination registry, we previously reported the association of plasma XOR activity with serum uric acid level [18]. In the present study, some of the methods used were the same as in that investigation and have reproduced relevant descriptions from that prior report in the present text.

### 2.2. Participants

Using the MedCity21 health examination registry, the final 200 sequential participants who participated in the lifestyle course of the advanced comprehensive medical examination, which was designed to check the status of lifestyle-related diseases, such as hypertension, diabetes, dyslipidemia, visceral obesity, hyperuricemia, atherosclerosis, and cerebrovascular disease, were selected. For our analysis, participants being treated with a xanthine oxidoreductase inhibitor (*n* = 4), or uricosuric (*n* = 1) or insulin (*n* = 1) agents, or with missing data (*n* = 1) were excluded. As a result, 193 participants were enrolled as subjects in the present cross-sectional study.

### 2.3. Physical and Laboratory Measurements

Information regarding height, body weight, smoking, and alcohol consumption habits, present and past illness, and use of medication was obtained. Body mass index (BMI) was calculated as weight in kilograms divided by the square of height in meters (kg/m^2^). DM was diagnosed when fasting plasma glucose was ≥126 mg/dl or 2-hour plasma glucose during a 75 g oral glucose tolerance test was ≥200 mg/dl, glycated hemoglobin A1c was ≥6.5%, or previous therapy for DM had been received [21]. Hypertension was defined as systolic blood pressure of ≥140 mmHg, diastolic blood pressure of ≥90 mmHg, or treatment for hypertension [22]. Dyslipidemia was defined as low-density lipoprotein cholesterol of ≥140 mg/dl, high-density lipoprotein cholesterol of ≤40 mg/dl, triglycerides of ≥150 mg/dl, or treatment for dyslipidemia [23]. Blood was drawn after an overnight fast. Biochemical parameters were analyzed using a standard laboratory method, and the remaining blood samples were stored at −80°C. Serum creatinine was measured using an enzymatic method. Estimated glomerular filtration rate (eGFR) was calculated using an equation designed for Japanese subjects, as previously described [24]. Glycated hemoglobin A1c (HbA1c) levels were estimated as National Glycohemoglobin Standardization Program equivalent values (%) using the conversion formula established by the Japan Diabetes Society [25]. Serum adiponectin levels were measured using a latex particle-enhanced turbidimetric immunoassay (Otsuka Pharmaceutical Co., Tokyo, Japan) [26]. Serum immunoreactive insulin (IRI) levels were measured with an electrochemiluminescence immunoassay (Roche Diagnostics K.K., Tokyo, Japan). HOMA-IR index was calculated according to the following formula: fasting IRI (lU/mL) × fasting plasma glucose (mg/dL)/405 [14, 15].

### 2.4. Measurement of Visceral and Subcutaneous Fat Areas

Using abdominal CT (Supria Grande, Hitachi, Ltd., Tokyo, Japan), we acquired a single 5 mm slice at the level of the umbilicus, as we previously described [18]. VFA and subcutaneous fat area (SFA) were automatically segmented, while VFA and SFA values were also automatically calculated using the fatPointer software package, ver.2 (Hitachi, Ltd., Tokyo, Japan). According to the manufacturer of this system, when using the same CT image, the reproducibility of both VFA and SFA measurements are 100%.

### 2.5. Plasma XOR Activity

Freshly frozen samples maintained at −80°C until the time of the assay were used to determine plasma XOR activity with the recently established method for assays of stable isotope-labeled [^13^C_2_,^15^N_2_] xanthine with liquid chromatography (LC)/triple quadrupole mass spectrometry (TQMS), as we previously described [16, 17]. Briefly, 100 *μ*L aliquots of plasma were purified using a Sephadex G25 column, then mixed with Tris buffer (pH 8.5) containing [^13^C_2_,^15^N_2_] xanthine as a substrate, nicotinamide adenine dinucleotide^+^, and [^13^C_3_,^15^N_3_] uric acid as an internal standard, with the mixtures then incubated at 37°C for 90 minutes. Subsequently, they were combined with methanol (500 *μ*L) and centrifuged at 2000 ×*g* for 15  minutes at 4°C. Supernatants were collected, transferred to new tubes, and dried using a centrifugal evaporator. The residues were reconstituted in 150 *μ*L of distilled water and filtered through an ultrafiltration membrane, then measurements were performed using LC/TQMS. Calibration standard samples were examined for the amount of [^13^C_2_,^15^N_2_] uric acid produced, which was calculated using a calibration curve, with XOR activities expressed as that amount (pmol/h/mL).

### 2.6. Statistical Analysis

For statistical analysis, serum adiponectin, IRI, HOMA-IR, and plasma XOR activity were logarithm-transformed (log) to follow a normal distribution. Multiple regression analyses were performed to determine whether HOMA-IR was independently associated with plasma XOR activity after adjustment using various clinical parameters, including VFA and adiponectin level. We did not select serum uric acid level as a covariate because uric acid is produced from xanthine by an XOR-catalyzed reaction. The nonlinearity of the effects of VFA, adiponectin, and HOMA-IR was included in the regression model because significant nonlinearity was detected in these associations with plasma XOR activity. In addition, we incorporated a 2-factor interaction term (*sex∗HOMA* − IR) to assess the effect of sex difference on the relationship between HOMA-IR and plasma XOR activity. Variance inflation factor (VIF) was calculated to estimate the multicollinearity of each predictor. The reliability of the final regression model was internally validated using the bootstrap method. One hundred fifty sets of bootstrap samples were generated by resampling the original data, and the amount of optimism was estimated to determine the degree of overfitting. The R software package (version 3.2.2, R Foundation for Statistical Computing, Vienna, Austria) and Statistical Package for the Social Sciences software package (PASW Statistics, version 22.0) were used for data analysis. All reported *p* values are 2 tailed and were considered to indicate statistical significance at *p* < 0.05.

## 3. Results

### 3.1. Clinical Characteristics of Subjects

The characteristics of the enrolled subjects are shown in Table 1. The mean values for age, alanine aminotransferase (ALT), eGFR, uric acid, and HbA1c were 56.8 ± 12.7 years, 20.8 ± 11.7 U/L, 76.8 ± 14.2 mL/min/1.73 m^2^, 5.5 ± 1.3 mg/dL, and 5.8 ± 0.5 %, respectively, while those for VFA, log HOMA-IR, and log plasma XOR activity were 76.8 ± 45.8 cm^2^, 0.14 ± 0.30, and 1.50 ± 0.44 pmol/h/mL, respectively. The number of subjects with DM, hypertension, and dyslipidemia were 25, 55, and 90, respectively, and the number of subjects receiving medication for DM, hypertension, and dyslipidemia were 6, 35, and 29, respectively.

### 3.2. Association of HOMA-IR with Plasma XOR Activity Independent of Clinical Factors including Adiposity and Adiponectin

**Figure 1:**
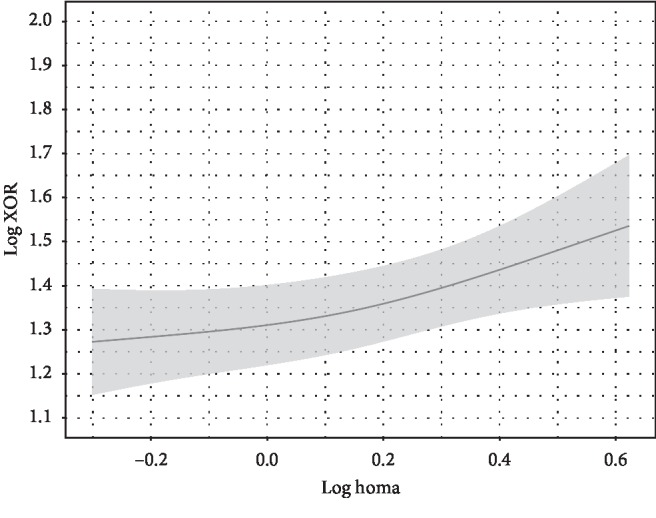
HOMA-IR and plasma XOR activity. HOMA-IR was independently associated with plasma XOR activity. Point estimates and the 95% confidence interval for these relationships are shown by a solid line and gray band, respectively. The value for plasma XOR activity is expressed as pmol/h/mL. Log plasma XOR activity was adjusted to the median values for age (56 years), sex (female), alcohol drinking habit (absence), smoking habit (absence), ALT (17 U/L), HbA1c (5.7 %), eGFR (76.8 mL/min/1.73 m^2^), VFA (69.8 cm^2^), and log adiponectin level (0.9345 *μ*g/mL).

**Figure 2:**
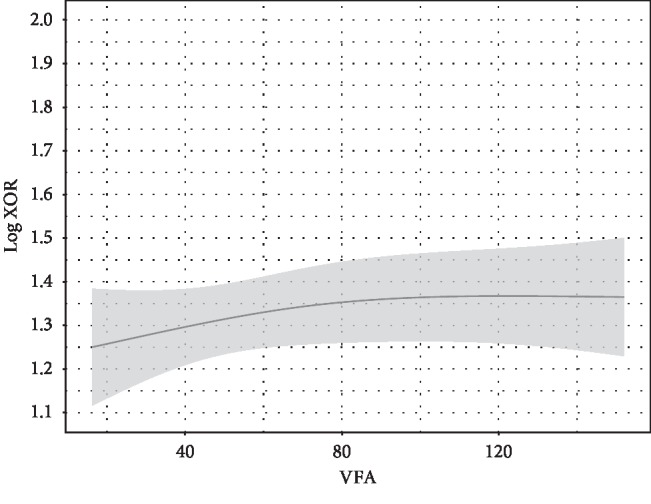
VFA and plasma XOR activity. VFA was not independently associated with plasma XOR activity. Point estimates and the 95% confidence interval for these relationships are shown by a solid line and gray band, respectively. Values for VFA and plasma XOR activity are expressed as cm^2^ and pmol/h/mL, respectively. Log plasma XOR activity was adjusted to the median values for age (56 years), sex (female), alcohol drinking habit (absence), smoking habit (absence), ALT (17 U/L), HbA1c (5.7 %), eGFR (76.8 mL/min/1.73 m^2^), log adiponectin level (0.9345 *μ*g/mL), and log HOMA-IR (0.1461).

**Figure 3:**
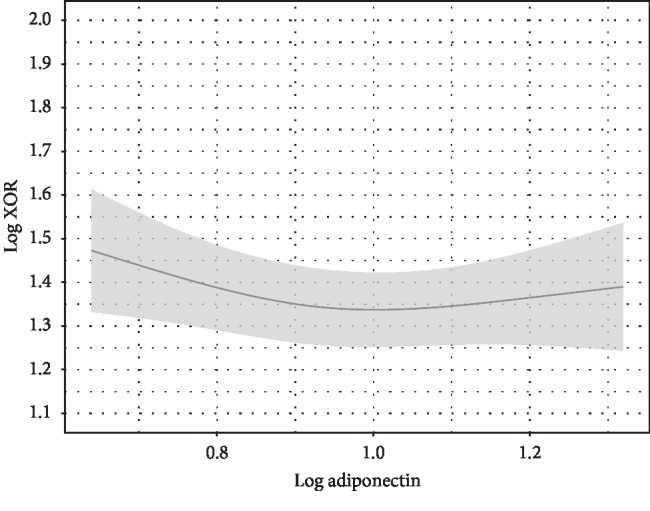
Adiponectin and plasma XOR activity. Adiponectin level was not independently associated with plasma XOR activity. Point estimates and the 95% confidence interval for these relationships are shown by a solid line and gray band, respectively. Values for adiponectin and plasma XOR activity are expressed as *μ*g/mL and pmol/h/mL, respectively. Log plasma XOR activity was adjusted to the median values for age (56 years), sex (female), alcohol drinking habit (absence), smoking habit (absence), ALT (17 U/L), HbA1c (5.7 %), eGFR (76.8 mL/min/1.73 m^2^), VFA (69.8 cm^2^), and log HOMA-IR (0.1461).

**Figure 4:**
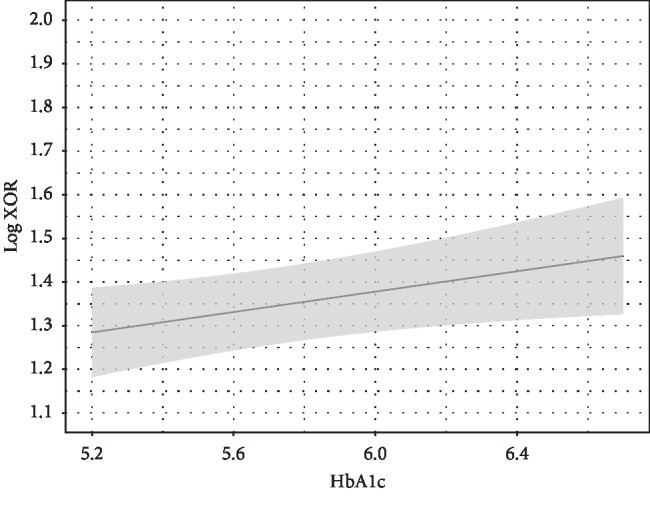
HbA1c and plasma XOR activity. HbA1c was independently associated with plasma XOR activity. Point estimates and the 95% confidence interval for these relationships are shown by a solid line and gray band, respectively. Values for HbA1c and plasma XOR activity are expressed as percent and pmol/h/mL, respectively. Log plasma XOR activity was adjusted to the median values for age (56 years), sex (female), alcohol drinking habit (absence), smoking habit (absence), ALT (17 U/L), eGFR (76.8 mL/min/1.73 m^2^), VFA (69.8 cm^2^), log adiponectin level (0.9345 *μ*g/mL), and log HOMA-IR (0.1461).

To examine whether HOMA-IR is independently associated with plasma XOR activity after adjustment for other confounding factors, including age, sex, alcohol drinking habit, smoking habit, ALT, HbA1c, eGFR, VFA, and adiponectin as covariates, multiple regression analyses were performed (Table 2). HOMA-IR, but not VFA or adiponectin, was significantly and independently associated with plasma XOR activity (Table 2; Figures 1–3). The “*sex∗HOMA* − IR” interaction was not significant (*p*=0.89), providing no evidence that sex difference has an effect on the relationship between HOMA-IR and plasma XOR activity. Sex and HbA1c, as well as ALT, were significantly associated with plasma XOR activity (Figure 4). Furthermore, the regression model was internally validated and the estimated optimism level was 0.042, indicating no evidence of overfitting. When VFA was replaced with VFA and SFA or BMI, the independent association of HOMA-IR, and no association of VFA and SFA or BMI, with plasma XOR activity was retained (Tables 3 and 4). The VIF value for each predictor was less than 5, indicating no multicollinearity between the variables (Tables 2–4). Also, when ALT was replaced with AST, similar results were obtained (data not shown).

## 4. Discussion

Findings in the present study demonstrated that HOMA-IR, but not VFA or serum adiponectin, has a significant independent association with plasma XOR activity. In previous reports, HOMA-IR, calculated using fasting insulin and glucose levels, has been well established as a simple and excellent index for insulin resistance; thus, it is considered to represent that mainly in the liver in healthy, obese, and diabetic individuals with compensatory hyperinsulinemia [14, 15, 27, 28]. The present results indicate that insulin resistance, but not visceral adiposity or reduction in serum adiponectin, might be independently associated with increased XOR activity, thus suggesting that development of insulin resistance resulting from increased visceral adiposity and/or reduced serum adiponectin may contribute to hyperuricemia by stimulating XOR activity.

In mice, XOR is highly expressed in the liver, intestines, and visceral fat [29], whereas in humans, it is known to be expressed in the liver and intestines but not in visceral fat [7, 30]. Consistent with that basic understanding, in the present multiple regression analysis, VFA, VFA and SFA, and BMI were not independently associated with plasma XOR activity (Tables 2–4; Figure 2). In addition, our findings support those of a previous report in which change in body weight was found to be not correlated with change in plasma XOR activity during weight loss program in obese subjects, whereas that was decreased after weight loss in those subjects [31]. Notably, XOR activity in the liver, in contrast to that in the intestines, is not determined by primary or genetic factors in hyperuricemia patients [10], indicating that secondary factors affect that activity in the liver. In the present study, HOMA-IR was significantly and positively associated with plasma XOR activity, independent of VFA and adiponectin level (Table 2; Figures 1–3). A previous animal study showed that elevated insulin directly enhanced XOR activity in the liver [32]. Furthermore, insulin resistance is considered to increase ribose-5-phosphate production by impairing the glycolysis pathway through reduced glyceraldehyde-3-phosphate dehydrogenase activity [33] and increase adenosine triphosphate degradation to adenosine monophosphate [34], suggesting that insulin resistance indirectly activates XOR activity in the liver via enhanced purine degradation. A previous clinical study also found that plasma XOR activity was significantly decreased after administration of metformin in patients with diabetes [35]. Thus, insulin resistance or hyperinsulinemia might contribute to increased XOR activity in the liver via a complex pathway, resulting in increased production and reduced renal excretion of uric acid. Because insulin resistance is known to be caused in part by hypoadiponectinemia associated with visceral fat accumulation [36, 37], previous findings of the association of serum adiponectin and serum uric acid levels [38] might reflect an association of insulin resistance with XOR activity in the liver.

Other important findings obtained in the present study include the association of HbA1c and gender with plasma XOR activity (Table 2, Figure 4). Several studies have shown that moderate glycemic control status is associated with higher serum uric acid levels [39, 40]. Other groups have demonstrated that blood XOR activity is higher in patients with DM [41, 42], whereas glycemic control status was shown to be positively correlated with blood XOR activity in patients with DM [42] or cardiovascular disease [43]. Furthermore, we previously reported that plasma XOR activity was significantly higher in hemodialysis patients with DM as compared with those without DM, and there was a positive correlation of glycemic control status and plasma XOR activity in hemodialysis patients with DM [44]. Of importance, an *in vitro* study found that glucose increases uric acid production by hepatocytes [45], while another study demonstrated that streptozotocin-induced diabetic rats have increased XOR activity in the liver without the release of other hepatic enzymes such as alanine aminotransferase [46]. Thus, hyperglycemia might directly activate hepatic XOR activity, resulting in a higher level of uric acid in DM patients.

Consistent with previous studies [47, 48] that reported higher hepatic XOR activity in male subjects and rats as compared with female subjects, we noted that male subjects in the present study had a significantly and independently higher level of plasma XOR activity than female subjects. Hepatic XOR activity is significantly increased after puberty, while it has been found to be significantly decreased following an orchiectomy in male rats, and hepatic XOR activity was shown to be significantly increased after an ovariectomy and following administration of testosterone in female rats [47]. Importantly, the “*sex∗HOMA* − IR” interaction was not significant in the present study, indicating that sex difference does not have an effect on the relationship between HOMA-IR and plasma XOR activity. Those previous studies along with the present results suggest that sex hormones modulate hepatic XOR activity, resulting in higher plasma XOR activity in male subjects as compared with female subjects, which may explain, at least in part, the generally higher uric acid level seen in male subjects.

In our previous study that also used the present MedCity21 health examination registry, we found that plasma XOR activity was positively and independently associated with serum uric acid level [18]. There are important differences between the present and that previous study. Although the final 200 sequential participants in the registry were selected and plasma XOR activity was measured using our novel highly sensitive assay in both, 2 participants treated with a sodium-glucose cotransporter 2 inhibitor and included in the present study were excluded from our previous database used for analysis, in order to eliminate findings from subjects taking a drug that can have effects on serum uric acid level. In addition, plasma XOR activity was used as a covariate and serum uric acid level as an independent variable in multivariable linear regression analyses to examine the association of plasma XOR activity with serum uric acid in our previous study. On the other hand, HOMA-IR was used as a covariate and plasma XOR activity as an independent variable in multivariable linear regression analyses performed in the present study in order to examine the association of insulin resistance with plasma XOR activity. Thus, the purposes, methods, and results differ between these investigations.

This was a cross-sectional study, thus even though relationships were explored in predictive terms, the results cannot be interpreted to show causal relationships. From this present study, the possibility that increased XOR activity contributes to increased insulin resistance could not be denied. Therefore, a longitudinal study is necessary to clarify the role of insulin resistance in regard to XOR activity.

## 5. Conclusions

Our results showed that insulin resistance is associated with plasma XOR activity in a manner independent of visceral adiposity and adiponectin level. Furthermore, they suggest that development of insulin resistance caused by increased visceral adiposity and/or reduced serum adiponectin may contribute to increased uric acid production by stimulating XOR activity.

## Figures and Tables

**Table 1:** Clinical characteristics of subjects (*n* = 193).

Age, years	56.8 ± 12.7
Males, *n*	92 (47.7%)
Alcohol drinking habit, *n*	63 (32.6%)
Smoking habit, *n*	39 (20.1%)
DM, *n*	25 (13.0%)
Hypertension, *n*	55 (28.5%)
Dyslipidemia, *n*	90 (46.6%)
Total bilirubin, mg/dL	0.7 ± 0.3
AST, U/L	22.6 ± 8.2
ALT, U/L	20.8 ± 11.7
eGFR, mL/min/1.73 m^2^	76.8 ± 14.2
Uric acid, mg/dL	5.5 ± 1.3
Fasting plasma glucose, mg/dL	103.4 ± 15.5
HbA1c, %	5.8 ± 0.5
BMI, kg/m^2^	22.9 ± 3.5
VFA, cm^2^	76.8 ± 45.8
SFA, cm^2^	141.4 ± 70.6
VFA and SFA, cm^2^	218.1 ± 96.7
Log [adiponectin, *μ*g/mL]	0.94 ± 0.20
Log [IRI, *μ*U/mL]	0.74 ± 0.27
Log [HOMA-IR]	0.14 ± 0.30
Log [XOR activity, pmol/h/mL]	1.50 ± 0.44

Data are presented as the mean ± standard deviation or *n* (%) for dichotomous variables. Adiponectin, IRI, HOMA-IR, and XOR activity were logarithm-transformed (Log) to achieve a normal distribution. Abbreviations: AST, aspartate aminotransferase; ALT, alanine aminotransferase; eGFR, estimated glomerular filtration rate; HbA1c, glycated hemoglobin; BMI, body mass index; VFA, visceral fat area; SFA, subcutaneous fat area; IRI, immunoreactive insulin; HOMA-IR, homeostatic model assessment of insulin resistance; XOR, xanthine oxidoreductase.

**Table 2:** Multiple regression analysis of factors associated with plasma XOR activity.

Independent variable	Percentile		Coefficient			*p* value	VIF
	25th	75th	Difference	95% LCI	95% UCI		
Age	47	67	0.033	−0.054	0.119	0.455	1.964
Sex (male = 1, female = 0)	0	1	0.099	0.006	0.192	0.037	1.390
Alcohol drinking habit (present = 1, absent = 0)	0	1	−0.070	−0.160	0.021	0.130	1.243
Smoking habit (present = 1, absent = 0)	0	1	0.078	−0.026	0.183	0.139	1.197
ALT	13.0	25.0	0.219	0.166	0.271	0.001	1.542
HbA1c	5.5	6.0	0.058	0.007	0.110	0.027	1.464
eGFRxct	66.3	86.5	0.060	−0.008	0.128	0.082	1.540
VFA	43.8	107.6	0.063	−0.034	0.160	0.426	2.330
Log adiponectin	0.813	1.041	−0.043	−0.108	0.021	0.079	1.888
Log HOMA-IR	−0.046	0.322	0.100	0.030	0.171	0.020	1.918

Abbreviations: XOR, xanthine oxidoreductase; ALT, alanine aminotransferase; HbA1c, glycated hemoglobin; eGFR, estimated glomerular filtration rate; VFA, visceral fat area; HOMA-IR, homeostatic model assessment of insulin resistance; LCI, lower confidence interval; UCI, upper confidence interval; VIF, variance inflation factor.

**Table 3:** Multiple regression analysis of factors associated with plasma XOR activity including VFA and SFA.

Independent variable	Percentile		Coefficient			*p* value	VIF
	25th	75th	Difference	95% LCI	95% UCI		
Age	47	67	0.052	−0.026	0.130	0.192	1.668
Sex (male = 1, female = 0)	0	1	0.110	0.015	0.206	0.024	1.520
Alcohol drinking habit (present = 1, absent = 0)	0	1	−0.065	−0.155	0.025	0.154	1.225
Smoking habit (present = 1, absent = 0)	0	1	0.073	−0.032	0.178	0.172	1.211
ALT	13.0	25.0	0.228	0.173	0.283	0.001	1.693
HbA1c	5.5	6.0	0.058	0.006	0.109	0.028	1.466
eGFR	66.3	86.5	0.062	−0.006	0.130	0.075	1.543
VFA and SFA	153.7	265.1	0.027	−0.050	0.104	0.498	2.535
Log adiponectin	0.813	1.041	−0.050	−0.112	0.012	0.072	1.778
Log HOMA-IR	−0.046	0.322	0.108	0.030	0.185	0.021	2.344

Abbreviations: XOR, xanthine oxidoreductase; ALT, alanine aminotransferase; HbA1c, glycated hemoglobin; eGFR, estimated glomerular filtration rate; VFA, visceral fat area; SFA, subcutaneous fat area; HOMA-IR, homeostatic model assessment of insulin resistance; LCI, lower confidence interval; UCI, upper confidence interval; VIF, variance inflation factor.

**Table 4:** Multiple regression analysis of factors associated with plasma XOR activity including BMI.

Independent variable	Percentile		Coefficient			*p* value	VIF
	25th	75th	Difference	95% LCI	95% UCI		
Age	47	67	0.057	−0.021	0.134	0.154	1.686
Sex (male = 1, female = 0)	0	1	0.109	0.018	0.201	0.020	1.380
Alcohol drinking habit (present = 1, absent = 0)	0	1	−0.064	−0.154	0.026	0.161	1.223
Smoking habit (present = 1, absent = 0)	0	1	0.075	−0.030	0.180	0.163	1.218
ALT	13.0	25.0	0.228	0.173	0.283	0.001	1.696
HbA1c	5.5	6.0	0.054	0.003	0.105	0.038	1.462
eGFR	66.3	86.5	0.057	−0.011	0.125	0.098	1.544
BMI	20.7	24.7	−0.007	−0.075	0.061	0.832	2.457
Log adiponectin	0.813	1.041	−0.062	−0.122	−0.003	0.054	1.726
Log HOMA-IR	−0.046	0.322	0.117	0.042	0.193	0.010	2.266

Abbreviations: XOR, xanthine oxidoreductase; ALT, alanine aminotransferase; HbA1c, glycated hemoglobin; eGFR, estimated glomerular filtration rate; BMI, body mass index; HOMA-IR, homeostatic model assessment of insulin resistance; LCI, lower confidence interval; UCI, upper confidence interval; VIF, variance inflation factor.

## Data Availability

The data analyzed during the study are not publicly available, in order to protect patient privacy, as it might be possible to identify the results of an individual patient from this limited group of patients.
